# Full Breit Hamiltonian in the Multiwavelets Framework

**DOI:** 10.1021/acs.jctc.3c01056

**Published:** 2024-01-01

**Authors:** Christian Tantardini, Roberto Di Remigio Eikås, Magnar Bjørgve, Stig Rune Jensen, Luca Frediani

**Affiliations:** † Hylleraas Centre, UiT The Arctic University of Norway, P.O. Box 6050 Langnes, N-9037 Tromsø, Norway; ‡ Department of Materials Science and NanoEngineering, Rice University, Houston, Texas 77005, United States; § Algorithmiq Ltd., Kanavakatu 3C, FI-00160 Helsinki, Finland

## Abstract

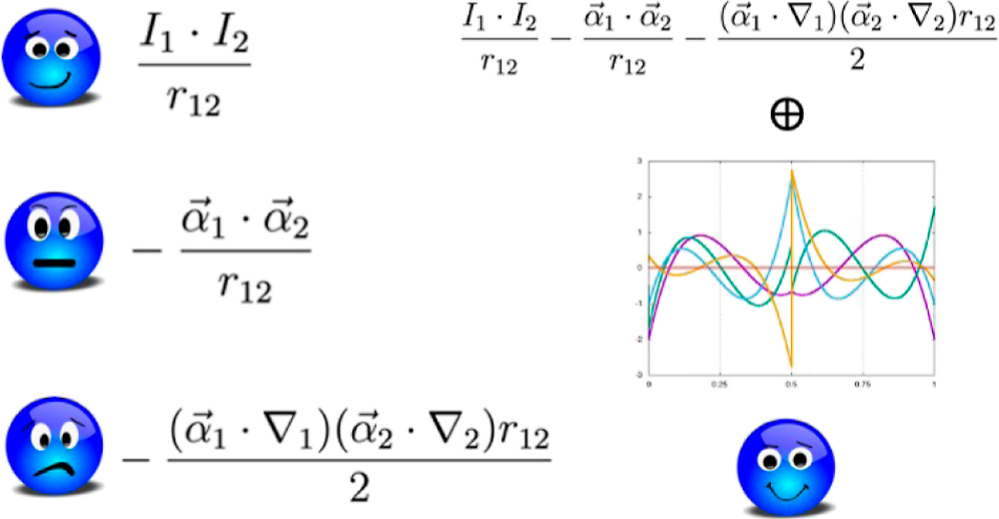

New techniques in core–electron spectroscopy are necessary to resolve the structures of oxides of *f*-elements and other strongly correlated materials that are present only as powders and not as single crystals. Thus, accurate quantum chemical methods must be developed to calculate core spectroscopic properties in such materials. In this contribution, we present an important development in this direction, extending our fully adaptive real-space multiwavelet basis framework to tackle the four-component Dirac-Coulomb-Breit Hamiltonian. We show that multiwavelets can reproduce one-dimensional grid-based approaches. They are however a fully three-dimensional approach which can later be extended to molecules and materials. Our multiwavelet implementation attained precise results irrespective of the chosen nuclear model, provided that the error threshold is tight enough and that the chosen polynomial basis is sufficiently large. Furthermore, our results confirmed that in two-electron species, the magnetic and Gauge contributions from *s*-orbitals are identical in magnitude and can account for the experimental evidence from *K* and *L* edges.

## 1 Introduction

Core–electron spectroscopies like X-ray photoelectron spectroscopy, X-ray absorption spectroscopy, and electron energy loss spectroscopy are powerful tools to investigate the electronic structure of transition-metal and rare-earth materials. For example, multilayered transition-metal carbides and carbonitrides M_*n*+1_AX_*n*_, where M is an early transition metal, A is an A-group element (mostly groups 13 and 14), X is C, or/and N and *n* is 1–3.^1^ These materials can be employed for energy storage systems, such as lithium-ion batteries,^1−5^ lithium-ion capacitors,^6^ aqueous pseudocapacitors,^7,8^ and transparent conductive films.^9^ Additionally, rare earths are contained in transparent conducting oxides which are considered the new frontier in the area of optoelectronics.^10−12^ These materials have the unique behavior of being both optically transparent and electrically conducting which makes them key components in many optoelectronic devices such as solar cells, flat panel displays, thin-film transistors, and light-emitting diodes.^10−12^

Unfortunately, their spectra are not straightforwardly interpretable due to relativistic effects. All relativistic effects, such as spin–orbit interactions, electron–electron interaction in the valence shell, and between core and valence electrons, will play a role in the core–electron spectra.^13−22^ A computational approach based on first-principles calculations that will take into account both relativity and electron correlation could help the interpretation of such spectra. A recent, promising approach in quantum chemistry is based on multiresolution analysis (MRA), by making use of multiwavelets (MWs).^23^ This method has gained momentum in recent years and has been applied to compute complete basis set limit results for energies and linear response properties of a large number of compounds both within Hartree–Fock (HF) and density functional theory (DFT).^24−28^ A variational treatment of relativistic effects into MRA will allow modeling the spectra of transition metal and rare-earth materials. An important step in this direction was presented to tackle the mean-field atomic and molecular Dirac-Coulomb problem in an adaptive, four-component MW basis.^29,30^ In such a model, the electrons are considered static charges where the average interaction between electrons is modeled with the Coulomb-like term only. This is the lowest-order relativistic approximation for the two-electron interaction, which disregards the magnetic interactions, such as spin-other-orbit and the retardation effects due to the finite speed of light. These effects are important and must be taken into account for a realistic modeling of core–electron spectroscopies. Therefore,^31−34^ the Breit interaction terms must be included.^35−39^ The Breit Hamiltonian adds two negative terms, called Gaunt and Gauge, respectively
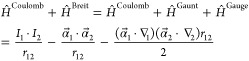
1

The first term in eq 1 is the nonrelativistic Coulombic interaction. The second term, called Gaunt, can be seen, in the nonrelativistic limit, as the scalar product between the curl of two spin orbitals: .^37^ α⃗ denotes a Cartesian vector collecting the 4 × 4 Dirac matrices α_*x*_, α_*y*_, and α_*z*_ (vide infra). When α⃗ acts on a four-component orbital, it couples its components, as detailed later on in this contribution. This means that the spin rotation of one electron on its axis generates a vector potential that will interact with the vector potentials generated by all other electrons present in the system,^37^ resulting in a scalar potential. Finally, the third term, called Gauge, describes the retardation effects due to the reciprocal interaction between the rotational vector fields (α_*i*_·∇_*i*_) of two electrons.^37^ These contributions cannot be neglected in systems that contain heavy or superheavy elements, especially in the calculation of core spectroscopic properties.^31−34^

In this contribution, we will present the adaptive MRA MW implementation of the *full* Breit interaction as a perturbative correction on top of a four-component Dirac-Coulomb-Hartree–Fock (DCHF) wave function. We will demonstrate the precision of our implementation by comparing ground-state energies of highly charged helium-like ions with increasing *Z*, X^(Z–2)+^, performed with our Python code, *VAMPyR* (Very Accurate Multiresolution Python Routines)^40^ with numerical radial integration in *GRASP*(41) and Gaussian basis set calculations with the*DIRAC*^42^ software.

## 2 Theory and Implementation

### 2.1 Multiresolution Analysis and Multiwavelets

MRA^43^ is constructed by considering a set of orthonormal functions called *scaling* functions ϕ_*i*_(*x*) supported on the interval [0,1]. They can be dilated and translated to obtain a corresponding basis in subintervals of [0,1]. The most common procedure is a dyadic subdivision, such that at scale *n*, there will be 2^*n*^ intervals defined by a translation index *l* = 0, 2^*n*^ – 1 such that the scaling functions in the *l*-th interval [*l*/2^*n*^, (*l* + 1)/2^*n*^] are obtained as
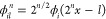
2Additionally, functions at subsequent scales are connected by the *two-scale relationships* which allow the scaling function at scale *n* to be obtained as a linear combination of scaling functions at scale *n* – 1.

This construction leads to a ladder of scaling spaces in a telescopic sequence that is dense in *L*^2^
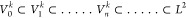
3

The *wavelet functions* are then obtained as the orthogonal complement of the scaling functions at scale *n* + 1 with respect to the ones at scale *n*.
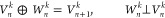
4

In the construction of Alpert,^44^ the scaling functions are a simple set of polynomials, and the wavelet functions are then piecewise polynomial functions. The possibility of constructing efficient algorithms, with precise error control, relies on the combination of several properties of such a construction. Here, it will suffice to say that the most important aspects concern the disjoint support of the basis, which enables function-based adaptivity, the vanishing moments of the wavelet functions, which guarantees fast decay of the representation coefficients, the nonstandard form of operators,^45^ which uncouples scales during operator application thus preserving adaptivity, the separated representation of integral kernels, which leads to low-scaling algorithms. The interested reader is referred to the available literature for details about these aspects.^23,44,46,47^

### 2.2 Mean-Field Two-Electron Operators on a Multiwavelet Basis

We will summarize the main methodological developments enabling the results in this contribution. We first recall that in a relativistic framework, molecular orbitals are vectors with four complex components. We will use indices:

- *u*, *w* ∈ {*x*, *y*, *z*} for Cartesian components,
- *p*, *q*, ... for occupied four-component orbitals,
- *A*, *B*, ... ∈ {1, 2, 3, 4} for orbital components.

Furthermore, Greek capital letters will be used for the four-component orbitals, and their lowercase counterparts will be used for the corresponding components
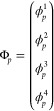
5The corresponding Hermitian conjugate (transposed and complex conjugate) orbital is
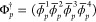
6with † denoting Hermitian conjugation and overline complex conjugation of a component.

To avoid confusion, we will also refer to the instantaneous electron interaction (first term in eq 1) as the *Coulomb* term, whereas we will use the terms *direct* and *exchange* to refer to the two parts of each term, arising from the Fermionic nature of the electrons.

For the Coulomb operator , the direct and exchange operators are straightforward and shown in eqs (7a) and (7b) in the Supporting Information, respectively. In practice, these operators are applied as convolutions. Efficient and accurate convolution with an integral operator is implemented in a separated representation (see ref (47) for details). We underline that the Coulomb part of the two-electron interaction in this framework is *diagonal*, in the sense that it is not coupling the four components of the spinor. In a Gaussian Type Orbital (GTO) framework, the exchange part would instead couple the four components of the spinor because the formalism is tied to the atomic orbital (AO) densities, thus generating an artificial coupling once the exchange operation is performed.^48^

We proceed similarly for the Gaunt operator . Note that the α⃗ appearing in the numerator are Cartesian vectors whose components are 4 × 4 antidiagonal block matrices
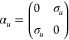
7with σ_*u*_, *u* ∈ {*x*, *y*, *z*}, the Pauli matrices. Applying α_*u*_ on a four-component orbital, in practice, reorders the components, possibly multiplied by a phase factor.

The two-electron energy for the Gaunt operator is thus
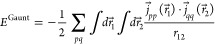
8
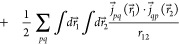
9where we have introduced the current density Cartesian vector, with components
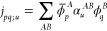
10to rewrite the expression more compactly. The corresponding mean-field, effective one-electron, direct and exchange operators are
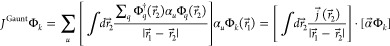
11a
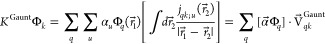
11b*j⃗* is the trace of the matrix collecting the orbital-pair current densities *j*_*pq*;*u*_.

The Gaunt direct and exchange operators use the same primitive as the Coulomb operators for convolution with the inverse-distance kernel. Thus:

1. Although the expressions for the Gaunt mean-field operators appear more complicated than those stemming from the Coulombic interaction, their computational load is only three times higher because each component of the α⃗ vector only has four nonzero elements.
2. For each Cartesian component, one can compute a “Gaunt potential” which is then multiplied by the α⃗-transformed orbital, exactly as for the Coulombic interaction.

Turning our attention to the gauge two-electron potential, we follow the suggestion of Sun et al.^49^ and rewrite it as
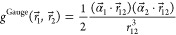
12
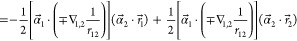
13where the sign/index pairs (−∇_1_ or + ∇_2_) can be chosen independently for each of the two terms, giving rise to *four* equivalent expression.

The energy expressions corresponding to each of the above forms can be considerably simplified by using integration by parts, thus avoiding the need for differentiating the inverse-distance kernel. However, of the four forms presented above, the energy expression obtained by choosing +∇_2_ in both terms of eq 13 is the most compact *and* computationally parsimonious
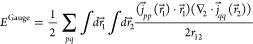
14
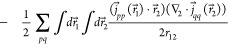
15
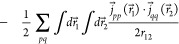
16
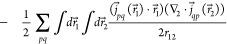
17
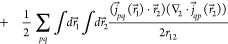
18
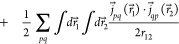
19

The former three terms are the direct contributions, and the latter three are the exchange contributions. The use of the inverse-distance kernel is the most significant advantage of this formulation since it is already an efficient and robust computational primitive in a MW basis. Note that the calculation of the divergence of the orbital current densities
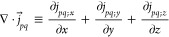
is both efficient and precise in a MW basis.^50^

Finally, we present the expressions for the direct and exchange Gauge mean-field operators
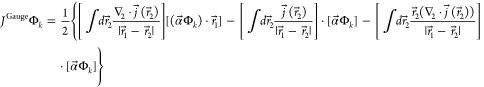
20a
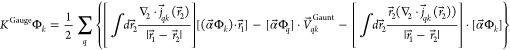
20b

All terms in both the direct and exchange operators are applied by using the inverse-distance integral operator only.

For completeness, we report also the expressions for the Gauge term when using the inverse-cube-distance form for the operator
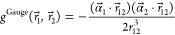
21The two-electron energy reads
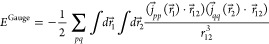
22
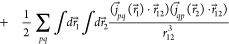
23

While this is arguably more compact than the sum of all six terms in the previous (eqs 14–19), it has two main disadvantages. First, it is harder to appreciate the physical content of the expression at a glance. Second, it requires the application of a different convolution operator. The latter point is apparent when looking at the expressions for the direct and exchange operators
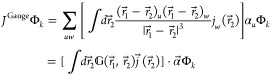
24a
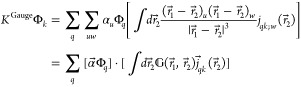
24b

The new convolution operator, , is a *matrix* convolution operator with six unique elements, each of which must be implemented by approximating the integral representation of the inverse-cube-distance kernel^51^ as a finite exponential sum^52^
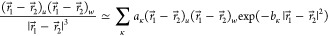
25Each term, though anisotropic, can be applied in each Cartesian direction separately. Coefficients and exponents in the sum are obtained similar to those for the inverse-distance convolution operator, see ref (47) for details. This form has been tested in our code, but it turned out to be less stable numerically and significantly more demanding computationally.

## 3 Computational Details

*DIRAC* calculations were performed using a nuclear point-charge model, and a threshold of 10^–7^ on the norm of the error vector (electronic gradient) was chosen as the convergence criterion for the SCF procedure. The chosen basis set for He, Ne^8+^, Ar^16+^, Kr^34+^, Xe^52+^, and Rn^84+^ was dyall-aug-cvqz.^38,53,54^ Furthermore, the calculations were performed using default settings for four-center integral screening and replacing (*SS*|*SS*) integrals by a simple Coulombic correction. In our MW implementation, it is not possible to perform such a correction because four-center integrals do not appear in the formalism. We investigated whether this could impact our perturbative/variational comparisons: with the full two-electron integral tensors, the total energy computed with *DIRAC* changes slightly, and computational cost increases *significantly*. However, the *relative error* with respect to both our implementation in *VAMPyR* and in *GRASP* was practically unaffected. This shows that the error is dominated by the intrinsic limitation of the basis set.

## 4 Results and Discussion

We present results for closed-shell, helium-like species: the core 1s-orbitals are doubly occupied, and our code explicitly enforces Kramers’ time-reversal symmetry (TRS),^55,56^ such that the four-component 1*s*^α^ is related to 1*s*^β^ by a quaternionic unitary transformation.^57^

In a mean-field treatment—e.g., HF and Kohn–Sham DFT—the Coulomb two-electron operator is replaced by the corresponding *Direct* and *Exchange* terms, indicated with *J* and *K*, respectively. Further inclusion of the Gaunt and Gauge interactions in eq 1 will result in additional *J*- and *K*-like terms. Making use of Kramers' TRS has a significant impact on the computational cost: the Coulombic interaction will encompass only the *direct* term, whereas the *exchange* one will be equal to zero. The Gaunt and Gauge interactions will give rise to both *direct* and *exchange* terms, but several contributions will either vanish or be identical to each other.

Previous work by Anderson et al.^30^ on *full* four-component Dirac-Coulomb relativistic calculations used smeared nuclear charge models.^58^ In particular for isolated atoms, they used the Fermi nuclear model.^58^ This was carried out to mitigate numerical issues by treating core orbitals with a point-charge model and improve precision. The Fermi model represents the nuclear charge using the Fermi–Dirac distribution for the nuclear charge density, introducing two parameters: the skin thickness and the half-charge radius. The former is set to 2.30 fm (2.30 × 10^–5^ Å) for all nuclei.^58^ The latter is the radius of a sphere containing half of the total nuclear charge. This parameter depends on the atomic mass of the nucleus *M*_N_, with one expression used when *M*_N_ ≤ 5 atomic mass units and another for *M*_N_ > 5.^58^ The Fermi model for the nuclear charge is smooth and, thus, more physically meaningful. Furthermore, it avoids singularities at the nuclei, in contrast to a point-like model. However, the results of Anderson et al.^30^ showed that the achieved precision of MW methods with respect to the grid-based approach available in *GRASP* decreases with increasing *Z*, even though a more physically motivated nuclear model was used.

**Figure 1:**
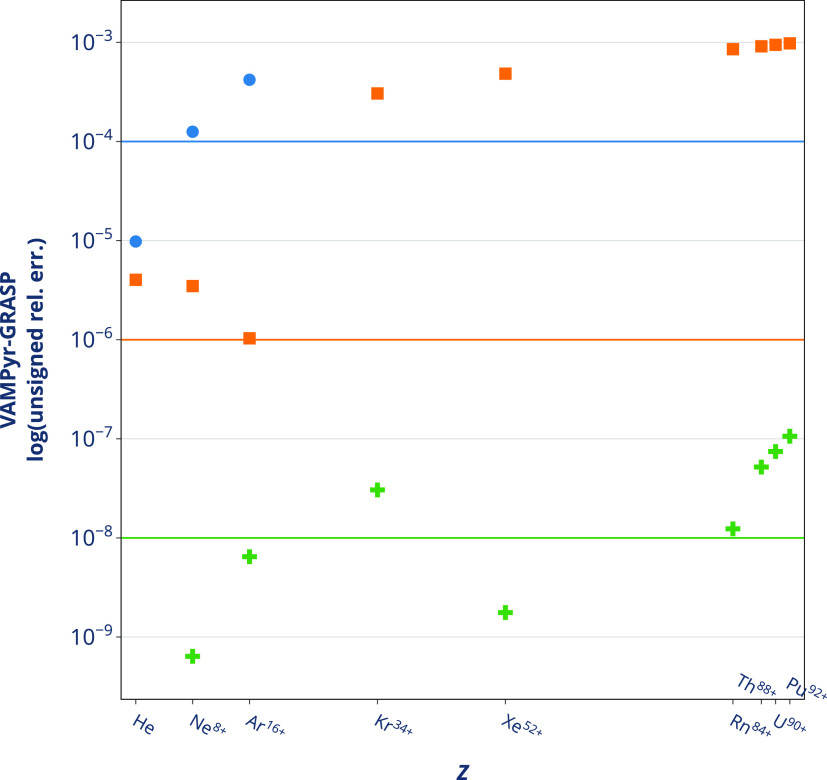
Logarithm of the unsigned relative error between the Dirac-Coulomb-Hartree–Fock ground-state energy calculations from *VAMPyR* and *GRASP*. All species are in the electronic configuration 1*s*^2^. The *VAMPyR* calculations were carried out with different choices of Legendre polynomial order *k* and tolerance ϵ: blue circle, *k* = 6, ϵ = 10^–4^; orange square, *k* = 8, ϵ = 10^–6^; green cross, *k* = 10, ϵ = 10^–8^. Both codes have used nuclear point charge model, as described in ref (58).

Our MW implementation in *VAMPyR* uses two parameters to tune the precision of the calculation: the tolerance, ε, and polynomial order, *k*. Furthermore, both point-charge and Fermi models are available for the nuclei. In order to validate our DCHF implementation and reassess the impact of the nuclear model, we performed DCHF calculations with a point-charge model and increasingly tighter precision settings. We report a comparison of our results with *GRASP* in Figure 1. The relative errors obtained at looser precision settings, as shown in Figure 1, are not consistent with the user-requested ϵ for heavy elements. The desired precision is user-selected through the settings for ϵ and *k* and should, in principle, be achieved, irrespective of the nuclear model. However, our results show that a point-charge nuclear model can reproduce grid-based results from *GRASP* only when a very tight tolerance is chosen, see Figure 1 and Table S1 in Supporting Information (SI). At the opposite end, SCF convergence could not be achieved for *k* = 6, ϵ = 10^–4^ for Kr^34+^ and heavier elements.

One possible explanation is the choice of point-like nuclear potential, which is nonphysical and not suitable for fully relativistic calculations but only for nonrelativistic ones. Thus, calculations with a point nucleus require a significantly tighter *tolerance* and consequently a higher polynomial order to achieve the same precision of grid-based results from *GRASP*.

After assessing the validity of our method for the DCHF equation, we developed the Gaunt and Gauge two-electron terms in the Breit Hamiltonian as a perturbative correction, as carried out in *GRASP*. The Gaunt term contains the vector operator α⃗ (it is a Cartesian vector of 4 × 4 matrices whose antidiagonal blocks are the Pauli matrices for the corresponding Cartesian direction). As we have previously mentioned in the *Introduction* section, it can be seen as the curl of a spin–orbital in the classical limit.^59^ α⃗ acting on a four-component orbital mixes its components to give the current density generated by the rotation of the spin around its axis.^59^

We first compared DCHF results from *DIRAC* with those obtained with *VAMPyR* at high precision (i.e., *k* = 10, and ϵ = 10^–8^), see Table S2 in Supporting Information. These results confirm and extend to the *full* four-component regime the observations of Jensen et al.: MWs can attain higher precision than large Gaussian atomic basis sets.^60^

**Figure 2:**
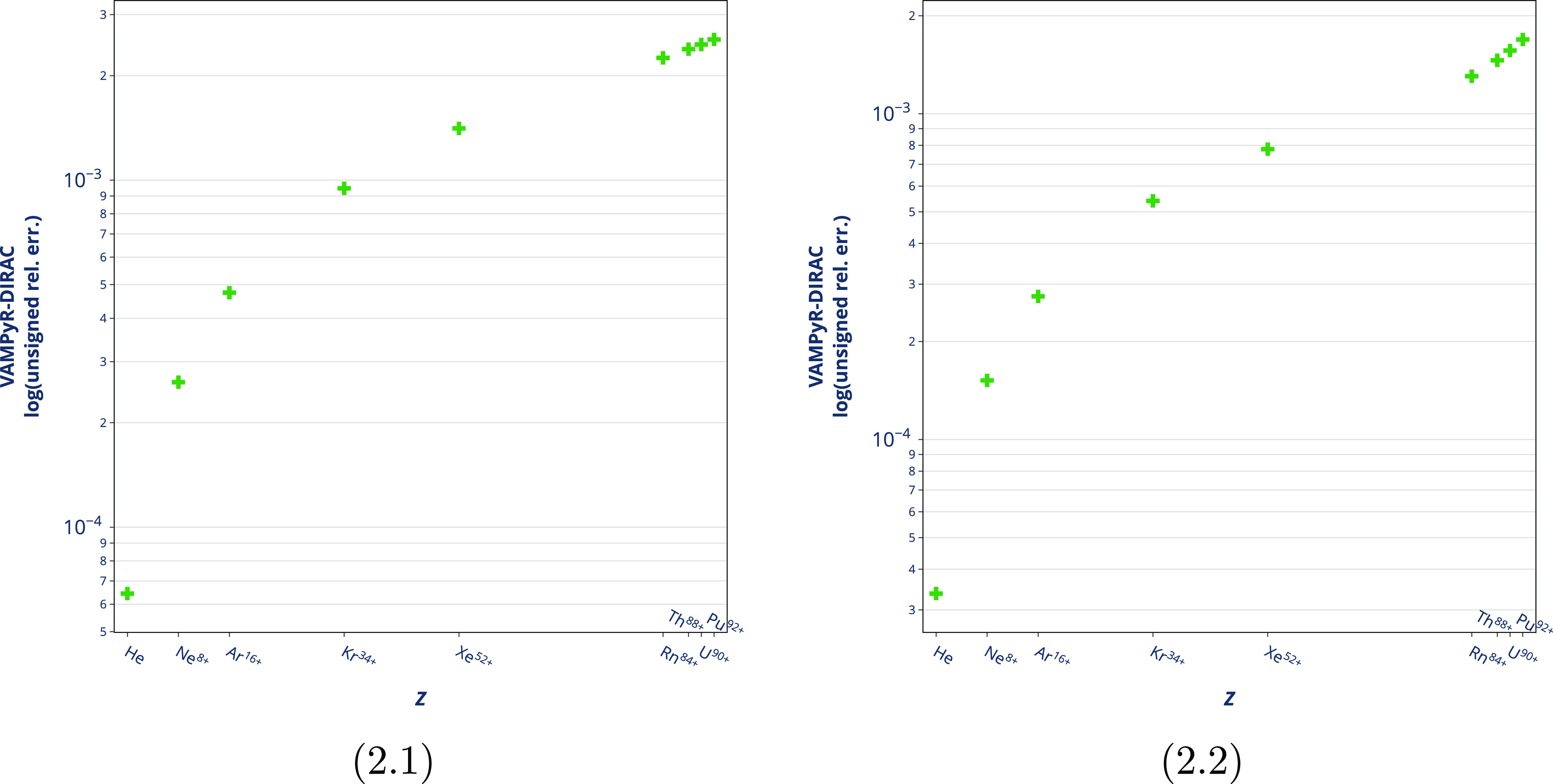
Comparison between the spin–orbit energies (left panel) and Gaunt terms (right panel) coming from *VAMPyR* and *DIRAC* for selected systems in the electronic configuration 1*s*^2^. The *y*-axis shows the logarithm of the unsigned relative difference between the *VAMPyR* and *DIRAC* results. The *VAMPyR* calculations were carried out with Legendre polynomial order *k* = 10 and tolerance ϵ = 10^–8^. All codes have used a nuclear point charge model, as described in ref (58).

Thereafter, we compared our perturbative Gaunt correction, implemented in *VAMPyR*, with the variational implementation available in the *DIRAC* code, see Figure 2. The inclusion of the Gaunt term in the variational self-consistent field procedure is not expected to significantly affect the ground state, as previously shown,^61^ and both results can be compared, see Figure 2. In fact, the logarithm of the unsigned relative errors for the spin–orbit energies, see Figure 2.1, and the Gaunt terms, see Figure 2.2, between *VAMPyR* and *DIRAC* have the same order of magnitude.

The perturbative Gauge correction only involves the inverse interelectronic distance kernel, as shown in (14)–(19), from which it is evident how the magnetic energy term arises as half of the Gaunt term since both the direct and exchange Gauge contributions (third and sixth terms) contain half of the Gaunt term.

**Figure 3:**
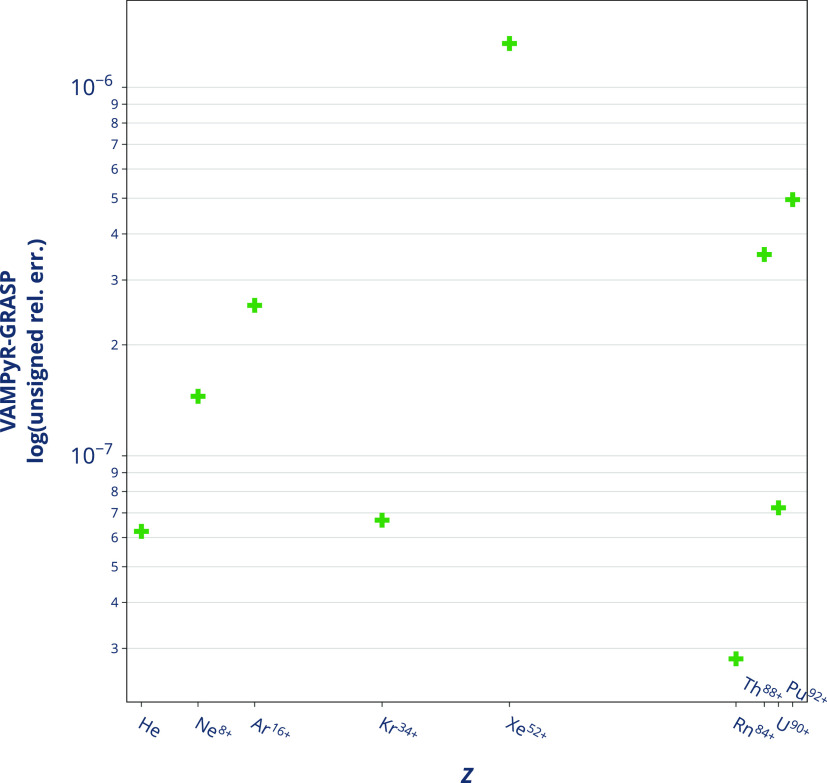
Comparison between the Breit perturbative corrections computed *VAMPyR* and *GRASP* for noble gases and actinides in the electronic configuration 1*s*^2^. The *y*-axis shows the logarithm of the unsigned relative difference between the *VAMPyR* and *GRASP* results. The *VAMPyR* calculations were carried out with Legendre polynomial order *k* = 10 and tolerance ϵ = 10^–8^. All codes have used a nuclear point charge model, as described in ref (58).

For the specific case of 1*s*^2^ systems, the terms involving a gradient in the Gauge energy (i.e., first eq 14, second eq 15, fourth eq 17, and fifth eq 18) are either zero or cancel each other out, up to the chosen numerical precision ε. Thus, the ratio between the Gauge term (*E*^Gauge^) and the magnetic interaction energy, which corresponds to half of the Gaunt term , should be one (i.e., identical magnetic and Gauge terms). This was verified comparing the Breit energy corrections from *VAMPyR* and *GRASP* results, see Figure 3 and Table S5 in the Supporting Information.

The *E*^Gauge^/*E*^Mag^ ratio was calculated previously using Gaussian AO basis sets for several atoms from *Z* = 9 to *Z* = 79.^49^ It was shown to range between 0.90 (fluorine) and 0.80 for *Z* > 56, converging asymptotically. In Table S5 of the Supporting Information, where we have considered 1*s*^2^ systems exclusively, we have obtained a unitary ratio between Gauge and magnetic terms. Furthermore, the magnitude of the Gauge term from our results in Table S5 in Supporting Information confirms what was previously found by Halbert et al.^62^ that in core–electron spectroscopy the Gauge term remains quite significant for the *K* and *L* edges, and it must be accounted for, especially for 1*s* to 2*s* transitions.^63^

## 5 Conclusions

We have shown that the four-component DCHF equations can be solved self-consistently with a fully adaptive MW basis irrespective of the chosen nuclear model, as required with Gaussian basis sets.^64,65^

The use of MRA with a MW basis to solve the KS-DFT equations allows one to separate model errors from discretization (i.e., basis set) errors, with the latter precisely quantifiable. Thus, the use of a MW basis provides fundamental insights to understand the range of applicability of KS-DFT with localized basis sets. This issue is especially relevant for four-component relativistic calculations on heavy elements where the description of the core–electrons is challenging due to the nature of the Dirac equation combined with the extremely high nuclear charge and a reduced availability of GTO bases.

We have shown that the DCHF ground state combined with the Breit Hamiltonian as a perturbative correction can reproduce grid-based calculations performed with *GRASP*. Albeit not performed in this work, the fully variational inclusion of the Gaunt and Gauge terms can be obtained by making use of the corresponding operator expressions (eqs 11a and 11b and 20a and 20b for Gaunt and Gauge, respectively). This has not been carried out for the current work both to simplify the comparison with the *GRASP* code and because of the excessive memory demands of the current pilot implementation. The latter is indeed the main challenge for future extensions to general molecular systems where the simplifications that enabled our results (Kramers' TRS and spherical symmetry of the 1*s* orbital) will no longer hold. Work is in progress in our group to overcome these hurdles.

The unitary *E*^Gauge^/*E*^Mag^ ratio for *s*-orbitals explains how neither Gaunt nor Gauge terms can be neglected for core–electron spectroscopy and explains the importance of considering both these terms when X-ray photoelectron spectra are calculated to fit the experimental ones.^62,63,66^ Our results confirm the validity of the MW approach for future development of core–electron spectroscopy to resolve the structures of oxides of *f*-elements and other strongly correlated systems.

## Supporting Information Available

All data generated or analyzed during this study (PDF)
