# Supplementary material for: Full Breit Hamiltonian in the Multiwavelets Framework
Source: J Chem Theory Comput. 2024 Jan 1;20(2):882–90. doi: 10.1021/acs.jctc.3c01056 (PMC10809419; doi:10.1021/acs.jctc.3c01056)
Supplement: Supplementary file 1 — ct3c01056_si_001.pdf [file ct3c01056_si_001.pdf]

Supporting Information
Future Perspective for Core-Electron Spectroscopy: Breit Hamiltonian in the Multiwavelets Framework

Christian Tantardini,[*,†,‡] Roberto Di Remigio Eikås,[†,¶] Magnar Bjørgve,[†] Stig Rune Jensen,[†] and Luca Frediani[*,†]

†*Hylleraas center, Department of Chemistry, UiT The Arctic University of Norway, PO Box 6050 Langnes, N-9037 Tromsø, Norway.*

‡*Department of Materials Science and NanoEngineering, Rice University, Houston, Texas 77005, United States of America*

¶*Algorithmiq Ltd., Kanavakatu 3C, FI-00160, Helsinki, Finland*

E-mail: christiantantardini@ymail.com; Luca.Frediani@uit.no

Derivation of mean-field two-electron operators

For completeness, we report here the general strategy for deriving the mean-field operators for any *multiplicative* two-electron operator.

The two-electron energy for an N-electron, single-determinant wavefunction is the sum

of direct and exchange components:

$$E_{\text{ee}} = \left\langle \frac{1}{2}\sum_{i=1}^{N}\sum_{j\neq i}^{N} g(\vec{r}_i,\vec{r}_j) \right\rangle_{\text{SD}} = \frac{1}{2}\sum_{pq}^{N}\int d\vec{r}_1 \int d\vec{r}_2 \Phi_p^\dagger(\vec{r}_1)\Phi_q^\dagger(\vec{r}_2) g(\vec{r}_1,\vec{r}_2)\Phi_p(\vec{r}_1)\Phi_q(\vec{r}_2) \quad (1)$$

$$- \frac{1}{2}\sum_{pq}^{N}\int d\vec{r}_1 \int d\vec{r}_2 \Phi_p^\dagger(\vec{r}_1)\Phi_q^\dagger(\vec{r}_2) g(\vec{r}_1,\vec{r}_2)\Phi_q(\vec{r}_1)\Phi_p(\vec{r}_2), \quad (2)$$

and the direct and exchange mean-field operators are obtained by functional differentiation with respect to a test function component $\overline{\varphi}_k^E(\vec{r})$:

$$\frac{\delta E_{\text{ee}}}{\delta \overline{\varphi}_k^E(\vec{r})} = (J-K)\Phi_k(\vec{r}_1) = \overbrace{\left[\int d\vec{r}_2 \sum_{q}^{N} \Phi_q^\dagger(\vec{r}_2) g(\vec{r}_1,\vec{r}_2)\Phi_q(\vec{r}_2)\right]\Phi_k(\vec{r}_1)}^{\text{Direct}} \quad (3)$$

$$\underbrace{- \sum_{q}^{N} \Phi_q(\vec{r}_1)\left[\int d\vec{r}_2 \Phi_q^\dagger(\vec{r}_2) g(\vec{r}_1,\vec{r}_2)\Phi_k(\vec{r}_2)\right]}_{\text{Exchange}} \quad (4)$$

Given these general formulas, it is enough to substitute the desired kernel $g(\vec{r}_1,\vec{r}_2)$ and carry out the algebra to obtain expressions for the energy and the operators for the direct and exchange terms.

As an example, consider the Coulomb operator $g^{Coulomb}(\vec{r}_1,\vec{r}_2) = \frac{I_1 \cdot I_2}{r_{12}}$. For the energy:

$$E^{Coulomb} = \frac{1}{2}\sum_{pq}\sum_{AB}\int d\vec{r}_1 \int d\vec{r}_2 \overline{\varphi}_p^A(\vec{r}_1)\varphi_p^A(\vec{r}_1)\frac{1}{r_{12}}\overline{\varphi}_q^B(\vec{r}_2)\varphi_q^B(\vec{r}_2) \quad (5)$$

$$- \frac{1}{2}\sum_{pq}\sum_{AB}\int d\vec{r}_1 \int d\vec{r}_2 \overline{\varphi}_p^A(\vec{r}_1)\varphi_q^A(\vec{r}_1)\frac{1}{r_{12}}\overline{\varphi}_q^B(\vec{r}_2)\varphi_p^B(\vec{r}_2), \quad (6)$$

while for the mean-field direct and exchange operators:

$$J^{Coulomb}\Phi_k = \left[\int d\vec{r}_2 \frac{\sum_q \Phi_q^\dagger(\vec{r}_2)\Phi_q(\vec{r}_2)}{|\vec{r}_1-\vec{r}_2|}\right]\Phi_k(\vec{r}_1) = \left[\int d\vec{r}_2 \frac{1}{|\vec{r}_1-\vec{r}_2|}\rho(\vec{r}_2)\right]\Phi_k \quad (7a)$$

$$K^{Coulomb}\Phi_k = \sum_q \Phi_q(\vec{r}_1)\left[\int d\vec{r}_2 \frac{\Phi_q^\dagger(\vec{r}_2)\Phi_k(\vec{r}_2)}{|\vec{r}_1-\vec{r}_2|}\right] = \sum_q \Phi_q V_{qk}^{Coulomb}, \quad (7b)$$

Additional data

Table 1: Comparison of *VAMPyR* and *GRASP* Dirac-Coulomb-Hartree-Fock ground-state energy calculations for noble gases and some actinides in electronic configuration $1s^2$. The *VAMPyR* calculations were done with different Legendre polynomial order k and tolerance ϵ. Both codes have used nuclear point charge model as described in Ref. 1. Energies are expressed in Ha.

		$(k=6, \epsilon=10^{-4})$		$(k=8, \epsilon=10^{-6})$		$(k=10, \epsilon=10^{-8})$	
Atom	*GRASP*	*VAMPyR*	Abs Rel. Error	*VAMPyR*	Abs Rel. Error	*VAMPyR*	Abs Rel. Error
He	-2.86181334	-2.861841357	$\lvert 9.790\rvert \cdot 10^{-06}$	-2.861824838	$\lvert 4.018\rvert \cdot 10^{-06}$	-2.86181334	$\lvert 2.064\rvert \cdot 10^{-10}$
Ne^{8+}	-93.98279960	-93.99457971	$\lvert 1.253\rvert \cdot 10^{-06}$	-93.98312622	$\lvert 3.475\rvert \cdot 10^{-06}$	-93.98279954	$\lvert 6.188\rvert \cdot 10^{-10}$
Ar^{16+}	-314.20016435	-314.3319089	$\lvert 4.193\rvert \cdot 10^{-04}$	-314.2004885	$\lvert 1.032\rvert \cdot 10^{-06}$	-314.20016638	$\lvert 6.463\rvert \cdot 10^{-09}$
Kr^{34+}	-1296.18692050	-	-	-1296.58252306	$\lvert 3.052\rvert \cdot 10^{-04}$	-1296.18695999	$\lvert 3.046\rvert \cdot 10^{-08}$
Xe^{52+}	-3003.18070400	-	-	-3004.63162311	$\lvert 4.831\rvert \cdot 10^{-04}$	-3003.18069870	$\lvert 1.763\rvert \cdot 10^{-09}$
Rn^{84+}	-8252.70532899	-	-	-8259.74602739	$\lvert 8.531\rvert \cdot 10^{-04}$	-8252.70522717	$\lvert 1.234\rvert \cdot 10^{-08}$
Th^{88+}	-9166.88303788	-	-	-9175.25052897	$\lvert 9.128\rvert \cdot 10^{-04}$	-9166.88256216	$\lvert 5.190\rvert \cdot 10^{-08}$
U^{90+}	-9651.39403060	-	-	-9660.50701703	$\lvert 9.442\rvert \cdot 10^{-04}$	-9651.39331292	$\lvert 7.436\rvert \cdot 10^{-08}$
Pu^{92+}	-10155.47184990	-	-	-10165.39209026	$\lvert 9.768\rvert \cdot 10^{-04}$	-10155.47077312	$\lvert 1.060\rvert \cdot 10^{-07}$

Table 2: Comparison of *VAMPyR*, *DIRAC* and *GRASP* Dirac-Coulomb-Hartree-Fock ground state energy calculations for noble gases and some actinides in electronic configuration $1s^2$. The *VAMPyR* calculations were done with Legendre polynomial order $k = 10$ and tolerance $\epsilon = 10^{-8}$. All codes have used nuclear point charge model as described in Ref. 1.

				Abs Relative Error		
Atom	*GRASP* / (Ha)	*VAMPyR* / (Ha)	*DIRAC* / (Ha)	*DIRAC-GRASP*	*VAMPyR-GRASP*	*VAMPyR-DIRAC*
He	-2.86181334	-2.86181334	-2.86179393	$\lvert 6.783\rvert \cdot 10^{-06}$	$\lvert 2.064\rvert \cdot 10^{-10}$	$\lvert 6.783\rvert \cdot 10^{-06}$
Ne^{8+}	-93.98279960	-93.98279954	-93.98280499	$\lvert 5.727\rvert \cdot 10^{-08}$	$\lvert 6.188\rvert \cdot 10^{-10}$	$\lvert 5.789\rvert \cdot 10^{-08}$
Ar^{16+}	-314.20016435	-314.20016638	-314.20035492	$\lvert 6.065\rvert \cdot 10^{-07}$	$\lvert 6.463\rvert \cdot 10^{-09}$	$\lvert 6.001\rvert \cdot 10^{-07}$
Kr^{34+}	-1296.18692050	1296.18695999	-1296.19345821	$\lvert 5.044\rvert \cdot 10^{-06}$	$\lvert 3.046\rvert \cdot 10^{-08}$	$\lvert 5.013\rvert \cdot 10^{-06}$
Xe^{52+}	-3003.18070400	-3003.18069870	-3003.23014898	$\lvert 1.646\rvert \cdot 10^{-05}$	$\lvert 1.763\rvert \cdot 10^{-09}$	$\lvert 1.647\rvert \cdot 10^{-05}$
Rn^{84+}	-8252.70532899	-8252.70522717	-8252.72444687	$\lvert 2.317\rvert \cdot 10^{-06}$	$\lvert 1.234\rvert \cdot 10^{-08}$	$\lvert 2.329\rvert \cdot 10^{-06}$
Th^{88+}	-9166.88303788	-9166.88256216	-9166.60598039	$\lvert 3.022\rvert \cdot 10^{-05}$	$\lvert 5.190\rvert \cdot 10^{-08}$	$\lvert 3.017\rvert \cdot 10^{-05}$
U^{90+}	-9651.39403060	-9651.39331292	-9650.85763397	$\lvert 5.558\rvert \cdot 10^{-05}$	$\lvert 7.436\rvert \cdot 10^{-08}$	$\lvert 5.551\rvert \cdot 10^{-05}$
Pu^{92+}	-10155.47184990	-10155.47077312	-10154.56584449	$\lvert 8.921\rvert \cdot 10^{-05}$	$\lvert 1.060\rvert \cdot 10^{-07}$	$\lvert 8.912\rvert \cdot 10^{-05}$

Table 3: Comparison between the spinorbit energies coming from *VAMPyR* and *DIRAC*, where the Gaunt term is implemented into the self consistently loop, for noble gases and actinides in electronic configuration $1s^2$. The *VAMPyR* calculations were done with Legendre polynomial order $k = 10$ and tolerance $\epsilon = 10^{-8}$. All codes have used nuclear point charge model as described in Ref. 1.

Atom	*DIRAC* / (Ha)	*VAMPyR* / (Ha)	Unsigned Rel. Error
He	$-9.179316 \cdot 10^{-01}$	$-9.179907 \cdot 10^{-01}$	$\lvert 6.439 \rvert \cdot 10^{-05}$
Ne^{8+}	$-4.396041 \cdot 10^{+01}$	$-4.397192 \cdot 10^{+01}$	$\lvert 2.6178 \rvert \cdot 10^{-04}$
Ar^{16+}	$-1.514799 \cdot 10^{+02}$	$-1.515517 \cdot 10^{+02}$	$\lvert 4.740 \rvert \cdot 10^{-04}$
Kr^{34+}	$-6.360600 \cdot 10^{+02}$	$-6.366624 \cdot 10^{+02}$	$\lvert 9.472 \rvert \cdot 10^{-04}$
Xe^{52+}	$-1.481682 \cdot 10^{+03}$	$-1.483771 \cdot 10^{+03}$	$\lvert 1.410 \rvert \cdot 10^{-04}$
Rn^{84+}	$-4.085169 \cdot 10^{+03}$	$-4.094366 \cdot 10^{+03}$	$\lvert 2.251 \rvert \cdot 10^{-03}$
Th^{88+}	$-4.538392 \cdot 10^{+03}$	$-4.549215 \cdot 10^{+03}$	$\lvert 2.385 \rvert \cdot 10^{-03}$
U^{90+}	$-4.778532 \cdot 10^{+03}$	$-4.790288 \cdot 10^{+03}$	$\lvert 2.460 \rvert \cdot 10^{-03}$
Pu^{92+}	$-5.028307 \cdot 10^{+03}$	$-5.041097 \cdot 10^{+03}$	$\lvert 2.544 \rvert \cdot 10^{-03}$

Table 4: Comparison between the Gaunt term coming from *VAMPyR*, which is implemented as added perturbation term, and *DIRAC*, which is implemented into the self consistently loop, for noble gases and actinides in electronic configuration $1s^2$. The *VAMPyR* calculations were done with Legendre polynomial order $k = 10$ and tolerance $\epsilon = 10^{-8}$. All codes have used nuclear point charge model as described in Ref. 1.

Atom	*DIRAC* / (Ha)	*VAMPyR* / (Ha)	Abs Rel. Error
He	$-6.377529 \cdot 10^{-05}$	$-6.377743 \cdot 10^{-05}$	$\lvert 3.367 \rvert \cdot 10^{-05}$
Ne^{8+}	$-1.210808 \cdot 10^{-02}$	$-1.210992 \cdot 10^{-02}$	$\lvert 1.519 \rvert \cdot 10^{-04}$
Ar^{16+}	$-7.397894 \cdot 10^{-02}$	$-7.399929 \cdot 10^{-02}$	$\lvert 2.751 \rvert \cdot 10^{-04}$
Kr^{34+}	$-6.173745 \cdot 10^{-01}$	$-6.177082 \cdot 10^{-01}$	$\lvert 5.405 \rvert \cdot 10^{-04}$
Xe^{52+}	$-2.161889 \cdot 10^{+00}$	$-2.163573 \cdot 10^{+00}$	$\lvert 7.790 \rvert \cdot 10^{-04}$
Rn^{84+}	$-9.650505 \cdot 10^{+00}$	$-9.663089 \cdot 10^{+00}$	$\lvert 1.304 \rvert \cdot 10^{-03}$
Th^{88+}	$-1.126074 \cdot 10^{+01}$	$-1.127718 \cdot 10^{+01}$	$\lvert 1.460 \rvert \cdot 10^{-03}$
U^{90+}	$-1.214446 \cdot 10^{+01}$	$-1.216346 \cdot 10^{+01}$	$\lvert 1.564 \rvert \cdot 10^{-03}$
Pu^{92+}	$-1.308523 \cdot 10^{+01}$	$-1.310736 \cdot 10^{+01}$	$\lvert 1.691 \rvert \cdot 10^{-03}$

Table 5: Comparison of the Breit correction to the Dirac-Coulomb-Hartree-Fock ground state energy calculations for He-like systems in electronic configuration $1s^2$ obtained with *VAMPyR* and *GRASP* . ($E^{Mag} = 1/2E^{Gaunt}$) and E^{Gauge} were performed with *VAMPyR*. The *VAMPyR* calculations were done with Legendre polynomial order $k = 10$ and tolerance $\epsilon = 10^{-8}$. Both codes have used nuclear point charge model as described in Ref. 1.

Atom	H^{Breit}_{GRASP} / (Ha)	H^{Breit}_{VAMPyR} / (Ha)	Unsigned Rel. Error	Mag. term	Gauge term	Gauge/Mag
He	$-6.37774337 \cdot 10^{-05}$	$-6.37774340 \cdot 10^{-05}$	$\|4.724\| \cdot 10^{-09}$	$-3.188872 \cdot 10^{-05}$	$-3.188872 \cdot 10^{-05}$	1.00
Ne^{8+}	$-1.21099236 \cdot 10^{-02}$	$-1.21099218 \cdot 10^{-02}$	$\|1.528\| \cdot 10^{-07}$	$-6.054961 \cdot 10^{-03}$	$-6.054961 \cdot 10^{-03}$	1.00
Ar^{16+}	$-7.39992715 \cdot 10^{-02}$	$-7.39992889 \cdot 10^{-02}$	$\|2.357\| \cdot 10^{-07}$	$-3.699964 \cdot 10^{-02}$	$-3.699964 \cdot 10^{-02}$	1.00
Kr^{34+}	$-6.17708182 \cdot 10^{-01}$	$-6.17708159 \cdot 10^{-01}$	$\|3.782\| \cdot 10^{-08}$	$-3.088541 \cdot 10^{-01}$	$-3.088541 \cdot 10^{-01}$	1.00
Xe^{52+}	$-2.16357594 \cdot 10^{+00}$	$-2.16357315 \cdot 10^{+00}$	$\|1.289\| \cdot 10^{-06}$	$-1.081787 \cdot 10^{+00}$	$-1.081787 \cdot 10^{+00}$	1.00
Rn^{84+}	$-9.66308942 \cdot 10^{+00}$	$-9.66308873 \cdot 10^{+00}$	$\|7.165\| \cdot 10^{-08}$	$-4.831544 \cdot 10^{+00}$	$-4.831544 \cdot 10^{+00}$	1.00
Th^{88+}	$-1.12771785 \cdot 10^{+01}$	$-1.12771760 \cdot 10^{+01}$	$\|2.145\| \cdot 10^{-07}$	$-5.638588 \cdot 10^{+00}$	$-5.638588 \cdot 10^{+00}$	1.00
U^{90+}	$-1.21634627 \cdot 10^{+01}$	$-1.21634591 \cdot 10^{+01}$	$\|2.940\| \cdot 10^{-07}$	$-6.081730 \cdot 10^{+00}$	$-6.081730 \cdot 10^{+00}$	1.00
Pu^{92+}	$-1.31073688 \cdot 10^{+01}$	$-1.31073635 \cdot 10^{+01}$	$\|4.069\| \cdot 10^{-07}$	$-6.553682 \cdot 10^{+00}$	$-6.553682 \cdot 10^{+00}$	1.00

References

(1) Visscher, L.; Dyall, K. DIRAC–FOCK ATOMIC ELECTRONIC STRUCTURE CALCULATIONS USING DIFFERENT NUCLEAR CHARGE DISTRIBUTIONS. *Atomic Data and Nuclear Data Tables* **1997**, *67*, 207–224.
